# Differential expression of adhesion molecules in sickle cell anemia and gut microbiome effect

**DOI:** 10.1007/s00277-023-05589-5

**Published:** 2023-12-28

**Authors:** Mariana Delgadinho, Luísa Veiga, Catarina Ginete, Brígida Santos, Armandina Miranda, Jocelyne Neto de Vasconcelos, Miguel Brito

**Affiliations:** 1 H&TRC - Health & Technology Research Center, ESTeSL - Escola Superior de Tecnologia da Saúde, Instituto Politécnico de Lisboa, Lisbon, Portugal; 2 Centro de Investigação em Saúde de Angola (CISA), Caxito, Bengo Angola; 3 Hospital Pediátrico David Bernardino (HPDB), Luanda, Angola; 4 Instituto Nacional de Saúde Doutor Ricardo Jorge (INSA), Lisbon, Portugal

**Keywords:** Sickle cell anemia, Adhesion molecules, Biomarkers, Hydroxyurea, Microbiome

## Abstract

Sickle cell anemia (SCA) causes a long-standing vascular inflammation state, leading to endothelial dysfunction and chronic overexpression of several adhesion molecules, which contributes to acute and constant vaso-occlusive (VOC) episodes. It has been demonstrated that hydroxyurea (HU) can reduce VOC events, organ damage, blood transfusions, and even the adhesion properties to endothelial cells of SCA subjects. Due to VOC episodes, these patients are also more susceptible to recurrent bacterial translocation and dysbiosis. Given this, our study aimed to uncover the interplay between adhesion molecules, gut microbiome, and hydroxyurea in a population of Angolan SCA children. Serum and fecal samples were obtained before and after HU treatment in 35 children. After HU, four of these adhesion molecules were significantly reduced: sE-selectin (*p* = 0.002), ADAMTS13 (*p* = 0.023), sICAM-1 (*p* = 0.003), and sVCAM-1 (*p* = 0.018). A positive correlation was observed between the number of neutrophils and sICAM-1, platelets, and sP-selectin, and also between leukocytes, sICAM-1, and sVCAM-1. Most taxa showing a significant correlation mainly belonged to the Clostridiales order. Specifically, from the *Clostridium* genera, the groups g19, g21, and g34 were all negatively correlated with HbF levels; g19, g21, and g24 positively correlated with leukocytes; g19 positively with neutrophils and sVCAM-1; and g34 positively with E- and P-selectin. *Serratia*, an opportunistic pathogen, was positively correlated with sE-selectin and sICAM-1 levels. Additionally, a negative correlation was observed between sP-selectin and *Bifidobacterium*. Research studies in this area could improve our understanding and contribute to finding new prognostic biomarkers to guarantee precise SCA patient stratification and predict severe complications.

**Supplementary Information:**

The online version contains supplementary material available at 10.1007/s00277-023-05589-5.

## Introduction

Sickle cell disease (SCD) is the most common inherited hematological disorder, accounting for over 300,000 births every year and responsible for significant morbidity and early mortality [1, 2]. On a global scale, the incidence of this disease is greatest in sub-Saharan Africa, where mortality rates for those under age 5 range from 50 to 80% [3]. Among the several types of SCD, HbSS, which is often described as sickle cell anemia (SCA), and hemoglobin SC disease (HbSC) are the most common [4]. The symptoms and complications associated with SCA are heterogeneous and can range from mild to very severe. Normally, a milder phenotype is associated with high levels of fetal hemoglobin (HbF) and co-inheritance of α-thalassemia, but these biomarkers explain only a small fraction of the observed phenotypic variability [5]. Finding new reliable biomarkers that explain this variability could assist clinicians in disease management and therapy response monitoring, allowing a more personalized approach for the SCA population [6].

Hydroxyurea (HU) is considered the gold standard therapeutic approach for SCA, having a multimodal effect by increasing the fetal hemoglobin level, reducing hemolysis and increasing NO bioavailability, modulating endothelial activation, and reducing neutrophil levels, which can contribute to the decrease of chronic inflammation [7]. Previous studies and clinical trials have consistently shown the efficacy of HU among children with SCD, by significantly reducing the vaso-occlusive (VOC) episodes, organ damage, acute chest syndrome, necessity for blood transfusions, and overall survival [8, 9].

The VOC episodes are unpredictable and extremely common in this population, being responsible for about 85% of SCD hospitalizations and emergency department visits[10]. Usually, the number of acute episodes tends to increase with age, and more than 50% of adults with SCD suffer from persistent chronic pain [11]. Leukocyte activation, oxidative stress, and ischemia/reperfusion are some factors specific to SCD that can trigger endothelial activation and lead to the development of VOC episodes [12]. Following the endothelial activation, nitric oxide bioavailability is impaired, and a variety of endothelial adhesion molecules are expressed (e.g., VCAM-1, selectins, ICAM-1), which will affect both the macro- and microvasculature [12, 13]. Even at steady state, SCD subjects have elevated levels of these adhesion molecules compared with controls, and it was reported a further increase during VOC episodes, suggesting a key involvement [4, 6, 10]. It has been demonstrated that HU is able to decrease the strength of erythrocytes adhesion in the vascular endothelial cell surface, modulating both VCAM-1 and ICAM-1 expression [14]. The hemoglobin polymerization in SCD leads to abnormal erythrocytes adhesion to the endothelium, a process that has been shown to be mediated by P- and E-selectins [15]. Moreover, a study demonstrated that levels of sVCAM-1 and thrombomodulin, markers of endothelial activation, were both more elevated in HbSS patients compared with HbSC patients and controls [16]. All things considered, these soluble adhesion molecules can act as markers of the presence and intensity of inflammation [17].

Recent studies have discovered that the intestinal microbiome is also critical in triggering VOC, mostly because of its role in regulating the aging of neutrophils [17]. It is suggested that SCD patients have a compromised intestinal barrier due to VOC episodes, being these individuals more susceptible to recurrent bacterial translocation and dysbiosis, which is likely contributing to the higher levels of circulating activated neutrophils [17, 18]. Furthermore, the gut microbiota was shown to influence the synthesis of endothelial adhesion molecules [19]. During inflammation, the intestinal epithelial barrier structure and function are disrupted, and in these inflammatory conditions, adhesion molecules are able to mediate the attachment of lymphocytes, neutrophils, and inflammatory cells to the endothelial cells [20]. All these observations suggest the existence of an important interplay between adhesion molecules, microbiome, and hydroxyurea. Although the molecular basis of SCD is well characterized, the complex mechanisms underlying this interplay have not been yet fully elucidated. The main importance of this area of research resides with the fact that new treatment approaches targeting gut modulation could be implemented, such as pro- and prebiotics, in order to reduce SCA symptomatology.

In this study, we measured potential cell adhesion and inflammatory biomarkers in Angolan SCA children, with the intention of investigating the relationship between these biomarkers with hematological parameters, hydroxyurea therapy, and gut microbiome.

## Materials and methods

### Subjects and sample collection

The study population consisted of Angolan SCA children aged 4–12 years, examined during periodical consultations at “Hospital Pediátrico David Bernardino” or “Hospital Geral do Bengo.” Whole blood samples and serums were collected from each SCA child for hematological and biochemical analysis. Additionally, fecal samples from each subject were collected in DNA/RNA Shield Fecal Collection tubes (Zymo Research).

### Hematological and biochemical analysis

Standard hematological laboratory assays were performed in CISA, which included complete blood count, hemoglobin, mean corpuscular volume (MCV), and mean corpuscular hemoglobin (MCH), which were determined using the XT-2000i Hematology Analyzer (Sysmex Corporation, Kobe, Japan) and HbF quantification by ion-exchange HPLC (Bio-Rad Variant II). Biochemical tests, such as lactate dehydrogenase (LDH), creatinine, aspartate aminotransferase (AST), and alanine aminotransferase (ALT) levels were performed using cobas C111 (Roche Diagnostics, Basel, Switzerland) and Mindray BA-88A (Mindray, Shenzhen, China).

### Serum biomarker assays

From the 80 serum samples received, 70 were eligible for analysis, which corresponded to 35 collected before initiation of HU treatment and 35 collected after 3 to 6 months of daily and continuous HU intake. The study includes sampling before and after the HU administration. A total of seven adhesion and inflammatory biomarkers were quantified, including the intracellular adhesion molecule-1 (ICAM-1), vascular cell adhesion molecule-1 (VCAM-1), platelet endothelial cell adhesion molecule-1 (sPECAM-1), ADAMTS13 (a disintegrin and metalloproteinase with a thrombospondin type 1 motif, member 13), thrombomodulin, and the platelet (P-selectin) and endothelial (E-selectin) selectins. These biomarkers were quantified using the MILLIPLEX® Human Cardiovascular Disease Magnetic Bead Panel 2 and 4 assays (Merck, Darmstadt, Germany). All procedures were carried out according to the manufacturer’s instructions, and samples were tested along with the quality controls and standard samples provided in the kits. After the protocol completion, the 96-well plate was placed onto Luminex MAGPIX system and analyzed using the xPONENT 4.2 software.

### 16S sequencing

DNA from fecal samples was extracted using ZymoBIOMICS™ DNA Miniprep Kit (Zymo Research), according to the manufacturer’s instructions. The NanoDrop One spectrophotometer and/or Qubit 3.0 fluorometer (Thermo Scientific) were used to quantify the DNA samples before and during 16S sequencing procedures. Preparation of libraries was performed following the 16S Metagenomic Sequencing Library Preparation Illumina document using primers for the V3-V4 hypervariable regions of the bacterial 16S rRNA gene and then sequenced on the NextSeq 550 (Illumina). The 16S rRNA data were analyzed using the EzBioCloud MTP pipeline and EzBioCloud 16S database PKSSU4.0 [21].

### Statistical analysis

Statistical analysis was performed using SPSS version 27 (IBM), biomarkers data were expressed as mean and standard deviation (M ± SD), and the means were compared using the Wilcoxon test. Spearman’s correlation analysis was used for correlation of parameters measured, and *p*-values less than 0.05 were considered statistically significant.

## Results

### Patients’ characteristics

A total of 35 HbSS patients were included in the study. These patients were children between 4 and 12 years; 47% were males and 53% females. At the time of the first sample collection, all the patients were naïve for hydroxyurea. After 3 to 6 months of daily and continuous HU intake, a second sample collection was performed during the follow-up consultations. As expected, several hematological parameters improved after HU administration, demonstrated by significant increases in total hemoglobin, HbF, MCV, and MCH, as well as reductions in white blood cells (WBC), neutrophils, reticulocytes, and total bilirubin, suggesting compliance with the treatment.

### Biomarker levels

**Fig. 1:**
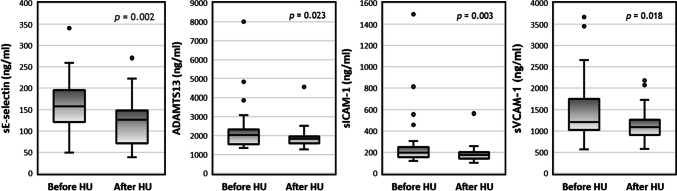
Boxplots representing significant variations between soluble biomarkers and HU administration. *p*-values calculated with non-parametric Wilcoxon paired test

The plasma levels of several soluble markers of adhesion or vascular inflammation were determined in SCA children both before and after hydroxyurea treatment (Table 1). We observed a significant decrease of sE-selectin (*p* = 0.002), ADAMTS13 (*p* = 0.023), sICAM-1 (*p* = 0.003), and sVCAM-1 (*p* = 0.018) after hydroxyurea, which is evident in the graphs from Fig. 1. However, no significant differences were found in the other four tested biomarkers: sP-selectin, sPECAM-1, and thrombomodulin.

**Table 1:** Assessment of adhesion and inflammatory biomarkers in SCA children both before and after hydroxyurea treatment

Biomarker (ng/ml)	Before HU (*n* = 35)	After HU (*n* = 35)	*p*-value
	Mean ± SD	Mean ± SD	
sE-selectin	148.65 ± 61.14	121.23 ± 56.97	0.002**
sP-selectin	137.47 ± 103.02	146.11 ± 104.21	0.399
sPECAM-1	2.61 ± 1.20	2.49 ± 1.01	0.554
sICAM-1	268.14 ± 249.81	183.66 ± 77.24	0.003**
sVCAM-1	1428.79 ± 746.39	1148.39 ± 414.60	0.018*
ADAMTS13	2234.73 ± 1229.42	1887.23 ± 549.65	0.023*
Thrombomodulin	4.89 ± 1.77	4.78 ± 1.73	0.374

Comparisons done with non-parametric Wilcoxon paired test

* and ** indicate *p*-value inferior to 0.05 and 0.01, respectively

Spearman’s rank correlation analysis was further applied to calculate the correlation between all the adhesion and inflammatory molecules of this study (Table 2). A total of nine significant correlations were identified. It was observed that sE-selection levels were positively correlated with sPECAM-1 and thrombomodulin and sP-selectin with sVCAM-1 and ADATMTS13. The only negative correlation was between sPECAM-1 and sICAM-1. The molecule sPECAM-1 was also positively correlated with thrombomodulin. sICAM-1 had a positive correlation with both sVCAM-1 and ADAMTS13. Lastly, sVCAM-1 and ADAMTS13 share also a positive correlation.

**Table 2:**
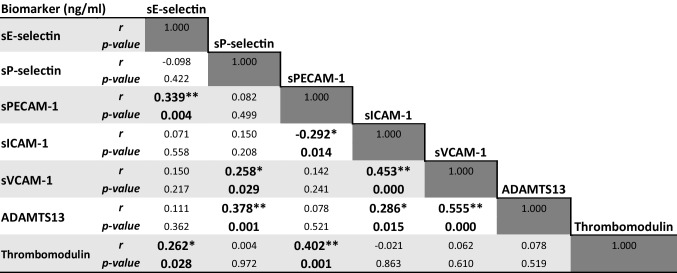
Spearman’s correlations between soluble biomarkers of SCA children (*N* = 70). * and ** indicate *p*-value inferior to 0.05 and 0.01, respectively

We also evaluated the relationship between these molecules’ levels to a variety of hematologic and biochemical measurements. Four significant positive correlations (Table 3) were identified between leukocytes with sICAM-1 and sVCAM-1, neutrophils and sICAM-1, and platelets and sP-selectin. The two negative correlations observed were hemoglobin with sVCAM-1 and creatinine with sICAM-1.

**Table 3:** Spearman’s correlations between soluble biomarkers and clinical parameters of SCA children (*N* = 70)

Biomarker (ng/ml)		Hb (g/dL)	HbF (%)	RBC (10^12^ l)	MCV (fL)	MCH (pg)	WBC (10^9^ l)	NEUT (10^9^ l)	PLAT (10^9^ l)	RETIC (%)	CREAT (mg/dL)	LDH (U/l)	AST (U/l)	ALT (U/l)
sE-selectin	*r*	0.012	− 0.123	0.104	− 0.146	− 0.165	0.108	0.140	− 0.002	0.151	− 0.123	− 0.016	− 0.151	− 0.075
	*p-value*	0.921	0.322	0.406	0.240	0.185	0.376	0.250	0.987	0.215	0.316	0.899	0.217	0.544
sP-selectin	*r*	0.079	0.100	− 0.093	0.114	0.115	0.120	0.215	0.348**	0.084	− 0.186	0.225	0.185	− 0.044
	*p-value*	0.511	0.415	0.450	0.352	0.352	0.320	0.071	0.003	0.484	0.121	0.060	0.122	0.717
sPECAM-1	*r*	0.075	− 0.009	0.017	− 0.022	− 0.010	− 0.050	0.012	0.158	0.010	− 0.047	− 0.193	− 0.146	− 0.215
	*p-value*	0.540	0.942	0.893	0.858	0.939	0.682	0.923	0.196	0.934	0.704	0.112	0.232	0.078
sICAM-1	*r*	− 0.198	− 0.215	0.012	− 0.107	− 0.191	0.387**	0.247*	− 0.047	0.230	− 0.306**	− 0.049	0.140	0.233
	*p-value*	0.097	0.077	0.923	0.382	0.118	0.001	0.038	0.695	0.054	0.010	0.686	0.244	0.052
sVCAM-1	*r*	− 0.334**	− 0.085	− 0.109	− 0.091	− 0.180	0.316**	0.135	− 0.153	0.195	− 0.211	− 0.010	− 0.093	− 0.043
	*p-value*	0.004	0.485	0.378	0.458	0.142	0.007	0.261	0.202	0.103	0.077	0.931	0.442	0.726
ADAMTS13	*r*	− 0.175	− 0.201	− 0.094	− 0.125	− 0.141	0.189	0.128	0.222	0.097	− 0.092	0.112	0.018	− 0.037
	*p-value*	0.144	0.098	0.444	0.306	0.251	0.114	0.289	0.063	0.420	0.447	0.352	0.883	0.761
Thrombomodulin	*r*	0.038	0.024	− 0.036	− 0.021	0.039	− 0.020	− 0.040	0.105	0.033	− 0.091	− 0.008	− 0.074	− 0.058
	*p-value*	0.757	0.846	0.775	0.867	0.753	0.869	0.744	0.389	0.789	0.455	0.945	0.545	0.636

* and ** indicate *p*-value inferior to 0.05 and 0.01, respectively

*Hb* total hemoglobin, *HbF* fetal hemoglobin, *RBC* red blood cells, *MCV* mean corpuscular volume, *MCH* mean corpuscular hemoglobin, *NEUT* neutrophils, *PLAT* platelets, *RETIC* reticulocytes, *CREAT* creatinine, *LDH* lactate dehydrogenase, *AST* aspartate aminotransferase, *ALT* alanine aminotransferase

### Correlations with the SCD microbiota

Based on the available metagenomics data, clinical results, and molecules quantification, a series of correlation analyses were performed. We observed that HbF levels were negatively correlated with a variety of bacterial genus: *Akkermansia*, *Mitsuokella*, *Clostridium_g21*, *Clostridium_g19*, *Clostridium_g34*, Ruminococcaceae_uc, *Oscillibacter*, *Sporobacter*, and *Ruminococcus_g2*. WBC count and neutrophils were both negatively correlated with *Intestinibacter* and positively correlated with 17 different bacteria: *Clostridium_g19*, *Clostridium_g34*, Prevotellaceae_uc, Lachnospiraceae_uc, *Ruminococcus_g4*, *Blautia*, *Lactococcus*, Pasteurellaceae_uc, *Prevotella*, *Ruminococcus_g5*, *Anaerostipes*, Bacillaceae_uc, *Faecalibacterium*, Streptococcaceae_uc, *Lachnobacterium*, *Lachnospira*, and *Veillonella*. Moreover, WBC was also positively correlated with *Clostridium_g21*, *Ruminococcus_g2*, Fusicatenibacter, Bacteroidaceae_uc, *Bacteroides*, *Agathobacter*, *Clostridium_g24*, *Enterococcus*, *Romboutsia*, *Streptococcus*, *Fusobacterium*, *Pseudoflavonifractor*, *Roseburia*, and *Subdoligranulum*.

**Fig. 2:**
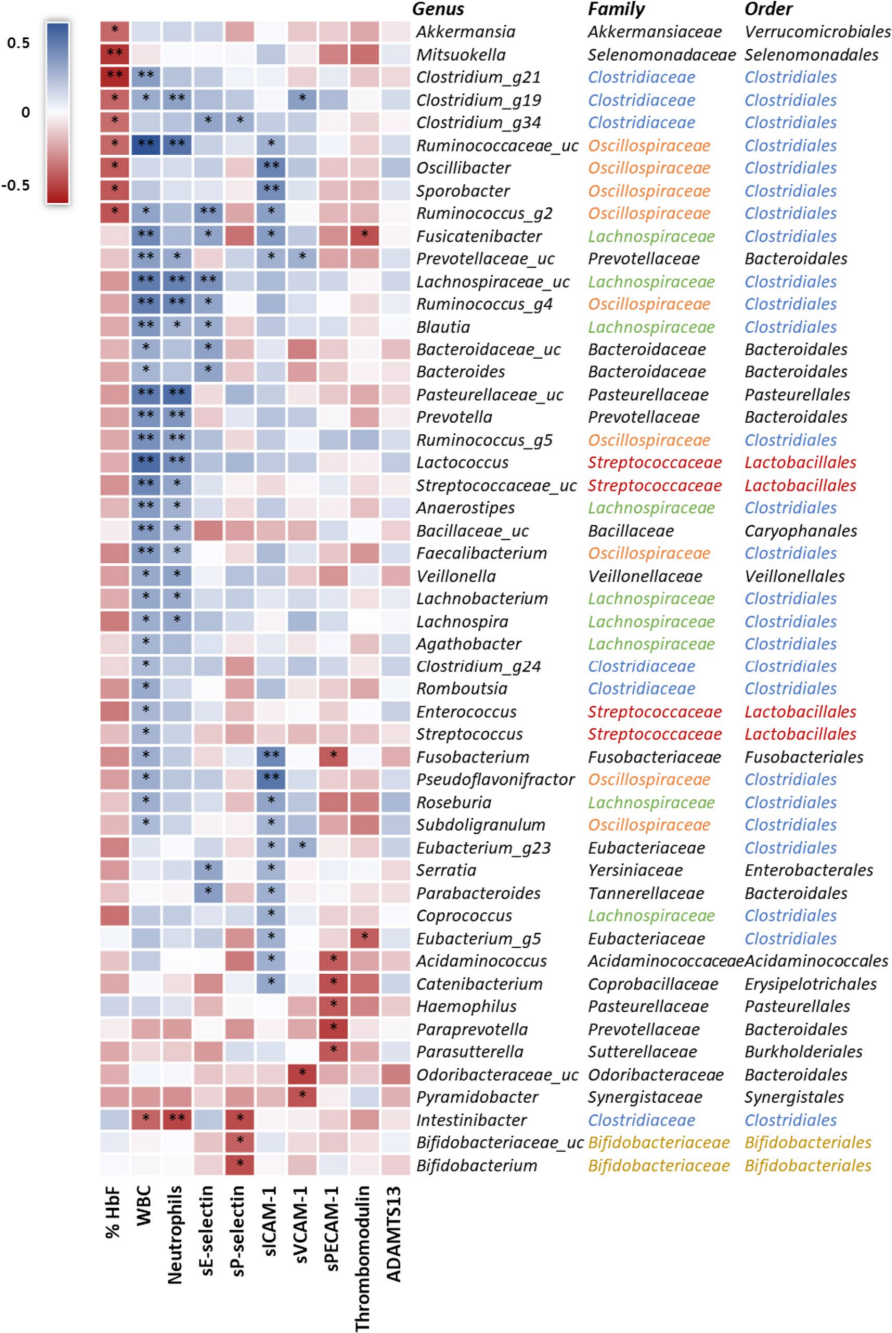
Correlation heat map (*N* = 70) between the SCA children’s gut microbiota and several biomarkers: HbF, WBC, neutrophils, sE-Selectin, sP-selectin, sICAM-1, sVCAM-1, sPECAM-1, thrombomodulin, and ADAMTS13. Spearman’s correlations were calculated only for the most abundant bacterial genera with a prevalence higher than 1%. Blue: positive correlations, red: negative correlations. *p < 0.05, **p < 0.01

Regarding the correlation results of the adhesion molecules (Fig. 2 and Table S1), sE-selectin was positively correlated with *Clostridium_g34*, *Ruminococcus_g2*, Fusicatenibacter, Lachnospiraceae_uc, *Ruminococcus_g4*, *Blautia*, Bacteroidaceae_uc, *Bacteroides*, *Serratia*, and *Parabacteroides*. sP-selectin had only one positive correlation with *Clostridium_g34* and three negative correlations with *Intestinibacter*, Bifidobacteriaceae_uc, and *Bifidobacterium*. As for sICAM-1, it was observed a positive correlation with Ruminococcaceae_uc, *Oscillibacter*, *Sporobacter*, *Ruminococcus_g2*, Fusicatenibacter, Prevotellaceae_uc, *Fusobacterium*, *Pseudoflavonifractor*, *Roseburia*, *Subdoligranulum*, *Eubacterium_g23*, *Serratia*, *Parabacteroides*, *Coprococcus*, *Eubacterium_g5*, *Acidaminococcus*, and *Catenibacterium*. The sVCAM-1 was only positively correlated with *Clostidium_g19*, Prevotellaceae_uc, and *Eubacterium_g23* and negatively correlated with Odoribacteraceae_uc and *Pyramidobacter*. The molecule sPECAM-1 had a negative correlation with *Fusobacterium*, *Acidaminococcus*, *Catenibacterium*, *Haemophilus*, *Paraprevotella*, and *Parasutterella*. Thrombomodulin was only negatively correlated with *Fusicatenibacter* and *Eubacterium_g5*. ADAMTS13 was the only molecule without any significant correlation with microbiome composition.

## Discussion

Through a single hemoglobin mutation, SCA has the capacity to shorten the life expectancy of millions of people globally. This disease causes a long-standing vascular inflammation state, leading to endothelial dysfunction and chronic overexpression of several adhesion molecules [22]. Disease severity is associated with abnormal erythrocyte adhesion to the vascular endothelium, thereby contributing to acute VOC episodes and chronic endothelial activation [23]. Studies have already demonstrated that sickle erythrocytes are more adherent than normal erythrocytes, which is correlated with disease severity and that HU therapy can significantly reduce the adhesion properties to endothelial cells of SCD subjects [24, 25].

But even when there is a clear clinical benefit from HU treatment, HbF response can vary significantly between patients [25]. Therefore, to confirm the patient’s clinical improvement, it is crucial to use additional biochemical laboratory parameters, not solely HbF levels. The impairment of NO bioavailability leads to the expression of soluble adhesion molecules, which can serve as biomarkers either of endothelial dysfunction or of inflammation with endothelial activation [26]. It has been demonstrated that the soluble molecules VCAM-1, ICAM-1, and E-selectin were associated with the risk of mortality in an SCD cohort and are also correlated with the severity of pulmonary hypertension, a clinical manifestation of endothelial dysfunction [26].

We noticed that the adhesion molecules with significant variations after HU were also the ones that exceeded the normal range, but we need to consider that this study population is neither adult nor healthy. Both sICAM-1 and sE-selectin levels in our study, independently of HU treatment, were in the same intervals of previous published data in healthy children [27], whereas sVCAM-1 highly surpassed this published normal range. Also, in a Brazilian adult HbSS population, without HU treatment, the median VCAM-1 value was 1492 ng/ml, a very similar result to our mean of 1429 ng/ml before therapy initiation [16]. Indeed, the literature describes higher levels of E-selectin, VCAM-1, and ICAM-1 in SCD patients compared to healthy controls and that hydroxyurea is expected to downregulate endothelial expression [25, 28]. And this effect was observed in our study, with significant decreases in these adhesion molecules after HU. One of the theories for the HU mechanism of action that has gained increasing support defends that HU leads to nitric oxide production, which has an essential role in regulating the expression of adhesion molecules, including the inhibition of VCAM-1 and ICAM-1 adhesion to the surface of endothelial cells [29].

ADAMTS13 was another soluble molecule that was significantly reduced after treatment. This molecule contributes to vaso-occlusion, but serum values of ADAMTS13 have been variable among SCD studies with some inconsistent findings [12]. Nevertheless, we found a study that reported increased levels of ADAMTS13 activity in SCD pediatric patients in comparison with a control group [30].

Another study described elevated levels of sP-selectin in SCD children and adolescents [31]. Moreover, P-selectin deficiency was proven to promote liver senescence in SCD mice [32]. However, the mean levels in our population were within the expected normal range of sP-selectin (50–150 ng/ml) [31] and without any significant differences after HU.

Increased thrombomodulin levels in circulation is a biomarker of endothelial cell injury that correlates with the severity of systemic vasculitis disorders and also predicts the risk of multiorgan failure in SCA patients [33]. In our study, we obtained thrombomodulin levels of 4.89 ± 1.77 before HU and 4.78 ± 1.73 after HU, higher than values reported in a study of SS population (3.5 ng/ml) [16], although still in the normal range of < 10 ng/ml [34].

As for PECAM-1, this molecule is known to act as a regulator of leukocyte trafficking, also having a role in the maintenance of endothelial cell junctional integrity [35]. We did not observe any significant difference before and after HU, and the amount of literature about this molecule’s effect on SCD is rather scarce.

A significant positive correlation was observed between levels of sVCAM-1 and sICAM-1, and also between sP-selectin and sVCAM-1, which is supported by previous studies and reflects the endothelial activation in these SCD patients [26, 29, 36]. It is common for adhesion molecules to show significant correlations with each other.

The sVCAM-1 levels were positively correlated with leukocytes and negatively with hemoglobin, an observation also made by other studies with SCD patients [6, 26]. One of these studies also reported a positive correlation between platelets and sP-selectin [26], and our results corroborate this finding. sP-selectin is released from platelets, being a biomarker for platelet activation indicated in cardiovascular events [37].

Moreover, we noticed that ICAM-1 was positively correlated with WBC. Interestingly, a study reported a positive correlation between adherent erythrocytes and WBC counts, and SCD subjects with clinically high WBC counts had also significantly greater erythrocytes adhesion to ICAM-1 in vitro [23]. Another remarkable finding in our results was the positive correlation between the number of neutrophils and sICAM-1. Neutrophils are known to express a variety of adhesion molecules on their surface, which are required for transendothelial migration, and it was observed that neutrophils of SCD patients are more able to adhere to fibronectin and endothelial monolayers than those of healthy individuals [38].

In previous papers from our research group, we have proved the existence of notable microbial differences between SCA patients and healthy controls [39] and have also reported that SCA children before HU treatment had a higher abundance of the pathogenic *Clostridium_g24*, whereas after HU, it was observed higher proportions of several beneficial bacteria, mostly short-chain fatty acids (SCFAs) producing species [40]. This last study was the first evidence of hydroxyurea’s positive effect on the gut microbiome. Given this, we sought to go further and analyze the influence of adhesion molecules in the microbiome of SCA patients.

The gut microbiota has already been shown to influence the synthesis of endothelial adhesion molecules [19]. For example, certain pathogenic species can lead to the upregulation of adhesion molecules followed by neutrophil recruitment, which can damage the healthy host tissues and negatively affect the commensal bacteria [41]. Moreover, a review has confirmed that five different soluble adhesion molecules are associated with the presence of sepsis, specifically, E-selectin, L-selectin, P-selectin, ICAM-1, and VCAM-1 [42].

In this work, we sought to compare the gut microbiome not only with adhesion molecules but also with some hematological parameters that we considered relevant for this population: leukocytes, neutrophils, and fetal hemoglobin. Looking at our most noteworthy results, a positive correlation was observed between three *Clostridium* bacteria (*Clostridium_g24*, *Clostridium_g21*, and *Clostridium_g19)* and leukocytes, biomarkers of infection when overexpressed. Moreover, the groups g21, g19, and g34 of the same bacteria were all negatively correlated with HbF levels, g19 positively with neutrophils and sVCAM-1 and g34 also positively with E- and P-selectin. The genus *Clostridium*, from the *Clostridiales* order, consists of over 100 species, many of which are known to cause disease in humans [43]. It is worth noting that most taxa showing a significant correlation with WBC, neutrophils, or HbF levels mainly belonged to the Clostridiales order. Studies have revealed that Clostridiales are more abundant in SCD patients than in healthy controls [44] and that this order is correlated with urinary 3-indoxyl sulfate levels [45]. Indoxyl sulfate is a compound that induces endothelial dysfunction, one central element implicated in cardiovascular morbidity and mortality [46]. Interestingly, this metabolite was found to be significantly higher in SCD individuals and correlates positively with circulating aged neutrophils [47].

Moreover, the four bacteria from the Streptococcaceae family (*Enterococcus*, *Streptococcus*, *Lactococcus*, Streptococcaceae_uc) were all positively correlated with WBC count and two of those genera (*Lactococcus* and Streptococcaceae_uc) had also a positive correlation with neutrophils levels. Streptococcaceae has been proven to be related to the development of bloodstream infections in hematologic malignancies and could be a strong predictor of infectious complications [48].

As for *Serratia*, this opportunistic pathogen was positively correlated with sE-selectin and sICAM-1 levels. Normally, it does not cause infections in healthy individuals, but certain conditions and therapies, that compromise patients immunologically, can cause susceptibility to *Serratia* colonization leading to a variety of infections and even death [49].

Another interesting result was the negative correlation between *Bifidobacterium* and Bifidobacteriaceae_uc to sP-selectin, which means that individuals with a higher abundance of these bacteria had lower sP-selectin values. The *Bifidobacterium* spp. is a dominant fraction of the human gut microbiota with beneficial anti-inflammatory effects, having several members of this genus commercially applied as probiotics [50]. *Intestinibacter* was another bacterium that correlated negatively with sP-selectin levels, but also negatively with leukocytes and neutrophils.

In SCA disease management, there is a high necessity to find and validate new prognostic biomarkers to guarantee precise patient stratification in relation to their risk for VOC complications and prevent the common acute episodes and other end-organ events [24]. Overall, our study contributes to this goal with interesting, detailed results about several adhesion molecules in an SCA pediatric population. Besides, this was also the first study to compare the SCA gut microbiome with these molecules. However, a larger cohort with similar sampling would be needed to deeper define and sustain our findings. Understanding these microbiota changes is rather challenging because of the tremendous complexity of the gut ecosystem and the impact of several external factors (e.g., diet, age, or BMI), which can possibly lead to a high risk of bias in microbiota research. Nevertheless, such investigations are still becoming increasingly important in order to establish new correlations in disease pathogenesis and find potential prognostic and therapeutic options.

### Supplementary Information

Below is the link to the electronic supplementary material.

Supplementary file1 (DOCX 44 KB)

## Data Availability

The datasets generated and/or analyzed during the current study could be available from the corresponding author on reasonable request.
