# Supplementary material for: Differential expression of adhesion molecules in sickle cell anemia and gut microbiome effect
Source: Ann Hematol. 2023 Dec 28;103(2):409–19. doi: 10.1007/s00277-023-05589-5 (PMC10799142; doi:10.1007/s00277-023-05589-5)
Supplement: Supplementary file 1 — Supplementary file1 (DOCX 44 KB) [file 277_2023_5589_MOESM1_ESM.docx]

# Supplementary Information

**Table S1–** Correlation table (N=70) between the SCA children’s gut microbiota and several biomarkers: % of fetal hemoglobin, leukocytes, neutrophils, sE-Selectin, sP-selectin, sICAM-1, sVCAM-1, sPECAM-1, thrombomodulin and ADAMTS13. Spearman’s correlations were calculated only for the most abundant bacterial genera with a prevalence higher than 1%. *p< 0.05, **p<0.01

|  |  | **HbF** | **WBC** | **NEUT** | **E-selectin** | **P-selectin** | **ICAM** | **VCAM** | **PECAM** | **Thromb** | **ADAMTS** |
| --- | --- | --- | --- | --- | --- | --- | --- | --- | --- | --- | --- |
| ***Acidaminococcus*** | Correlation Coefficient | -0.067 | 0.180 | 0.042 | 0.044 | -0.212 | ,265^*^ | 0.039 | -,270^*^ | -0.126 | -0.060 |
|  | p-value | 0.584 | 0.132 | 0.727 | 0.737 | 0.099 | 0.037 | 0.762 | 0.035 | 0.334 | 0.641 |
| ***Agathobacter*** | Correlation Coefficient | -0.031 | ,246^*^ | 0.232 | 0.115 | -0.001 | 0.098 | -0.001 | 0.060 | -0.071 | 0.139 |
|  | p-value | 0.800 | 0.039 | 0.051 | 0.376 | 0.995 | 0.448 | 0.991 | 0.647 | 0.585 | 0.281 |
| ***Akkermansia*** | Correlation Coefficient | -,248^*^ | 0.130 | 0.155 | 0.155 | -0.125 | 0.052 | -0.060 | -0.051 | -0.120 | 0.000 |
|  | p-value | 0.040 | 0.280 | 0.198 | 0.232 | 0.332 | 0.691 | 0.644 | 0.697 | 0.355 | 0.998 |
| ***Anaerostipes*** | Correlation Coefficient | -0.092 | ,323^**^ | ,259^*^ | 0.134 | -0.026 | 0.187 | 0.004 | 0.016 | -0.074 | 0.120 |
|  | p-value | 0.450 | 0.006 | 0.029 | 0.302 | 0.843 | 0.146 | 0.974 | 0.903 | 0.569 | 0.353 |
| ***Bacillaceae_uc*** | Correlation Coefficient | 0.011 | ,323^**^ | ,259^*^ | -0.193 | -0.137 | -0.094 | -0.099 | 0.135 | 0.038 | -0.043 |
|  | p-value | 0.930 | 0.006 | 0.029 | 0.136 | 0.287 | 0.465 | 0.446 | 0.299 | 0.772 | 0.740 |
| ***Bacteroidaceae_uc*** | Correlation Coefficient | -0.100 | ,273^*^ | 0.215 | ,303^*^ | -0.082 | 0.106 | -0.203 | -0.053 | 0.025 | -0.078 |
|  | p-value | 0.413 | 0.021 | 0.072 | 0.018 | 0.525 | 0.412 | 0.114 | 0.687 | 0.845 | 0.548 |
| ***Bacteroides*** | Correlation Coefficient | -0.126 | ,246^*^ | 0.204 | ,296^*^ | -0.057 | 0.160 | -0.139 | -0.054 | 0.022 | -0.007 |
|  | p-value | 0.300 | 0.039 | 0.088 | 0.020 | 0.658 | 0.214 | 0.282 | 0.682 | 0.867 | 0.954 |
| ***Bifidobacteriaceae_uc*** | Correlation Coefficient | 0.091 | 0.024 | 0.046 | -0.068 | -,261^*^ | 0.106 | -0.026 | -0.056 | -0.010 | 0.075 |
|  | p-value | 0.456 | 0.841 | 0.702 | 0.605 | 0.041 | 0.411 | 0.843 | 0.667 | 0.938 | 0.564 |
| ***Bifidobacterium*** | Correlation Coefficient | 0.053 | 0.030 | 0.038 | -0.041 | -,301^*^ | 0.029 | -0.077 | 0.097 | 0.006 | -0.045 |
|  | p-value | 0.664 | 0.802 | 0.756 | 0.752 | 0.018 | 0.826 | 0.550 | 0.455 | 0.965 | 0.728 |
| ***Blautia*** | Correlation Coefficient | -0.118 | ,334^**^ | ,253^*^ | ,275^*^ | -0.051 | 0.198 | 0.123 | 0.125 | -0.015 | 0.052 |
|  | p-value | 0.335 | 0.004 | 0.033 | 0.032 | 0.691 | 0.124 | 0.340 | 0.336 | 0.911 | 0.686 |
| ***Catenibacterium*** | Correlation Coefficient | -0.120 | 0.054 | -0.012 | -0.175 | 0.081 | ,290^*^ | 0.073 | -,296^*^ | -0.233 | 0.156 |
|  | p-value | 0.326 | 0.654 | 0.922 | 0.176 | 0.530 | 0.022 | 0.573 | 0.021 | 0.071 | 0.225 |
| ***Clostridium_g19*** | Correlation Coefficient | -,254^*^ | ,274^*^ | ,306^**^ | 0.228 | 0.190 | 0.056 | ,310^*^ | 0.227 | 0.030 | 0.139 |
|  | p-value | 0.035 | 0.021 | 0.009 | 0.077 | 0.138 | 0.663 | 0.014 | 0.078 | 0.816 | 0.281 |
| ***Clostridium_g21*** | Correlation Coefficient | -,370^**^ | ,316^**^ | 0.208 | 0.185 | 0.069 | 0.074 | -0.044 | 0.092 | -0.062 | -0.029 |
|  | p-value | 0.002 | 0.007 | 0.082 | 0.154 | 0.595 | 0.568 | 0.735 | 0.478 | 0.634 | 0.821 |
| ***Clostridium_g24*** | Correlation Coefficient | -0.030 | ,236^*^ | 0.181 | 0.193 | -0.156 | 0.147 | 0.202 | 0.150 | -0.003 | 0.164 |
|  | p-value | 0.810 | 0.048 | 0.132 | 0.136 | 0.226 | 0.255 | 0.115 | 0.249 | 0.983 | 0.202 |
| ***Clostridium_g34*** | Correlation Coefficient | -,237^*^ | 0.172 | 0.207 | ,293^*^ | ,265^*^ | 0.179 | 0.179 | 0.024 | -0.034 | 0.026 |
|  | p-value | 0.050 | 0.151 | 0.083 | 0.022 | 0.037 | 0.163 | 0.163 | 0.854 | 0.794 | 0.842 |
| ***Coprococcus*** | Correlation Coefficient | -0.229 | 0.190 | 0.182 | 0.115 | 0.141 | ,268^*^ | 0.086 | -0.041 | -0.041 | 0.049 |
|  | p-value | 0.058 | 0.113 | 0.128 | 0.378 | 0.274 | 0.035 | 0.508 | 0.756 | 0.752 | 0.707 |
| ***Enterococcus*** | Correlation Coefficient | -0.212 | ,241^*^ | 0.213 | 0.113 | -0.079 | 0.018 | 0.034 | -0.042 | 0.047 | 0.169 |
|  | p-value | 0.081 | 0.043 | 0.075 | 0.387 | 0.542 | 0.887 | 0.793 | 0.750 | 0.719 | 0.189 |
| ***Eubacterium_g23*** | Correlation Coefficient | -0.196 | 0.110 | 0.050 | 0.151 | -0.023 | ,251^*^ | ,265^*^ | 0.007 | -0.051 | 0.166 |
|  | p-value | 0.107 | 0.361 | 0.681 | 0.246 | 0.862 | 0.049 | 0.038 | 0.958 | 0.695 | 0.198 |
| ***Eubacterium_g5*** | Correlation Coefficient | 0.062 | 0.214 | 0.130 | 0.181 | -0.164 | ,260^*^ | 0.046 | -0.036 | -,264^*^ | 0.114 |
|  | p-value | 0.615 | 0.073 | 0.278 | 0.162 | 0.203 | 0.041 | 0.725 | 0.782 | 0.040 | 0.379 |
| ***Faecalibacterium*** | Correlation Coefficient | -0.182 | ,332^**^ | ,239^*^ | 0.045 | -0.023 | 0.232 | 0.111 | -0.062 | -0.159 | 0.129 |
|  | p-value | 0.134 | 0.005 | 0.045 | 0.731 | 0.860 | 0.070 | 0.390 | 0.636 | 0.222 | 0.319 |
| ***Fusicatenibacter*** | Correlation Coefficient | -0.023 | ,375^**^ | 0.233 | ,296^*^ | -0.227 | ,320^*^ | 0.191 | -0.169 | -,293^*^ | 0.027 |
|  | p-value | 0.851 | 0.001 | 0.050 | 0.020 | 0.076 | 0.011 | 0.137 | 0.192 | 0.022 | 0.836 |
| ***Fusobacterium*** | Correlation Coefficient | -0.177 | ,267^*^ | 0.186 | -0.030 | 0.100 | ,377^**^ | 0.050 | -,269^*^ | 0.054 | -0.111 |
|  | p-value | 0.146 | 0.024 | 0.120 | 0.820 | 0.442 | 0.003 | 0.697 | 0.036 | 0.679 | 0.392 |
| ***Haemophilus*** | Correlation Coefficient | 0.158 | 0.155 | 0.115 | -0.096 | 0.033 | 0.041 | -0.110 | -,276^*^ | -0.192 | -0.056 |
|  | p-value | 0.195 | 0.197 | 0.341 | 0.460 | 0.798 | 0.750 | 0.396 | 0.031 | 0.138 | 0.667 |
| ***Intestinibacter*** | Correlation Coefficient | 0.188 | -,253^*^ | -,315^**^ | 0.194 | -,305^*^ | 0.027 | 0.015 | -0.047 | -0.152 | -0.007 |
|  | p-value | 0.121 | 0.034 | 0.007 | 0.135 | 0.016 | 0.837 | 0.910 | 0.721 | 0.243 | 0.957 |
| ***Lachnobacterium*** | Correlation Coefficient | -0.115 | ,284^*^ | ,274^*^ | 0.141 | 0.178 | 0.103 | 0.143 | 0.104 | -0.010 | 0.094 |
|  | p-value | 0.349 | 0.016 | 0.021 | 0.277 | 0.167 | 0.424 | 0.266 | 0.424 | 0.938 | 0.468 |
| ***Lachnospira*** | Correlation Coefficient | -0.208 | ,268^*^ | ,288^*^ | 0.213 | 0.145 | 0.026 | 0.240 | 0.136 | 0.043 | 0.060 |
|  | p-value | 0.087 | 0.024 | 0.015 | 0.099 | 0.262 | 0.843 | 0.060 | 0.296 | 0.742 | 0.641 |
| ***Lachnospiraceae_uc*** | Correlation Coefficient | -0.156 | ,416^**^ | ,397^**^ | ,355^**^ | 0.163 | 0.232 | 0.154 | 0.150 | 0.029 | 0.149 |
|  | p-value | 0.202 | 0.000 | 0.001 | 0.005 | 0.205 | 0.070 | 0.231 | 0.250 | 0.826 | 0.247 |
| ***Lactococcus*** | Correlation Coefficient | -0.112 | ,460^**^ | ,366^**^ | 0.212 | 0.243 | 0.187 | 0.179 | 0.000 | 0.016 | 0.183 |
|  | p-value | 0.359 | 0.000 | 0.002 | 0.101 | 0.057 | 0.146 | 0.163 | 0.997 | 0.903 | 0.155 |
| ***Mitsuokella*** | Correlation Coefficient | -,347^**^ | -0.004 | 0.050 | 0.051 | 0.060 | 0.192 | 0.007 | -0.196 | -0.229 | 0.099 |
|  | p-value | 0.004 | 0.974 | 0.678 | 0.699 | 0.641 | 0.134 | 0.955 | 0.130 | 0.076 | 0.446 |
| ***Odoribacteraceae_uc*** | Correlation Coefficient | -0.111 | 0.064 | 0.058 | -0.065 | -0.036 | -0.041 | -,322^*^ | -0.114 | -0.054 | -0.204 |
|  | p-value | 0.366 | 0.594 | 0.632 | 0.621 | 0.782 | 0.750 | 0.011 | 0.384 | 0.678 | 0.112 |
| ***Oscillibacter*** | Correlation Coefficient | -,272^*^ | 0.146 | 0.136 | 0.177 | -0.058 | ,388^**^ | 0.200 | -0.060 | -0.053 | 0.214 |
|  | p-value | 0.024 | 0.223 | 0.258 | 0.171 | 0.657 | 0.002 | 0.119 | 0.645 | 0.682 | 0.094 |
| ***Parabacteroides*** | Correlation Coefficient | -0.069 | 0.034 | 0.026 | ,317^*^ | -0.064 | ,263^*^ | 0.017 | 0.031 | -0.014 | -0.013 |
|  | p-value | 0.575 | 0.776 | 0.827 | 0.013 | 0.624 | 0.039 | 0.896 | 0.812 | 0.912 | 0.918 |
| ***Paraprevotella*** | Correlation Coefficient | 0.012 | -0.121 | -0.144 | 0.034 | -0.161 | 0.022 | -0.121 | -,324^*^ | -0.006 | 0.030 |
|  | p-value | 0.920 | 0.314 | 0.230 | 0.795 | 0.212 | 0.866 | 0.348 | 0.011 | 0.965 | 0.814 |
| ***Parasutterella*** | Correlation Coefficient | -0.122 | -0.027 | -0.053 | -0.148 | 0.133 | 0.118 | 0.040 | -,277^*^ | -0.116 | 0.114 |
|  | p-value | 0.316 | 0.825 | 0.659 | 0.254 | 0.303 | 0.361 | 0.758 | 0.031 | 0.374 | 0.376 |
| ***Pasteurellaceae_uc*** | Correlation Coefficient | -0.149 | ,416^**^ | ,456^**^ | -0.023 | 0.249 | 0.176 | 0.001 | -0.060 | -0.119 | -0.036 |
|  | p-value | 0.223 | 0.000 | 0.000 | 0.859 | 0.051 | 0.171 | 0.994 | 0.649 | 0.363 | 0.781 |
| ***Prevotella*** | Correlation Coefficient | -0.134 | ,350^**^ | ,333^**^ | -0.057 | 0.105 | 0.206 | 0.187 | 0.031 | -0.128 | 0.011 |
|  | p-value | 0.271 | 0.003 | 0.005 | 0.664 | 0.415 | 0.108 | 0.145 | 0.813 | 0.326 | 0.931 |
| ***Prevotellaceae_uc*** | Correlation Coefficient | -0.065 | ,315^**^ | ,281^*^ | -0.042 | 0.164 | ,260^*^ | ,263^*^ | -0.129 | -0.133 | 0.126 |
|  | p-value | 0.593 | 0.007 | 0.018 | 0.745 | 0.202 | 0.041 | 0.039 | 0.322 | 0.307 | 0.328 |
| ***Pseudoflavonifractor*** | Correlation Coefficient | -0.142 | ,270^*^ | 0.200 | 0.187 | -0.032 | ,430^**^ | 0.136 | -0.048 | -0.076 | 0.138 |
|  | p-value | 0.246 | 0.023 | 0.094 | 0.148 | 0.803 | 0.000 | 0.292 | 0.715 | 0.559 | 0.286 |
| ***Pyramidobacter*** | Correlation Coefficient | -0.141 | -0.149 | -0.171 | -0.041 | -0.153 | -0.095 | -,287^*^ | 0.016 | 0.147 | -0.105 |
|  | p-value | 0.246 | 0.214 | 0.154 | 0.757 | 0.236 | 0.462 | 0.024 | 0.905 | 0.260 | 0.415 |
| ***Romboutsia*** | Correlation Coefficient | -0.162 | ,280^*^ | 0.147 | 0.042 | -0.130 | 0.218 | -0.003 | -0.050 | -0.122 | 0.062 |
|  | p-value | 0.182 | 0.018 | 0.221 | 0.745 | 0.315 | 0.089 | 0.981 | 0.705 | 0.348 | 0.635 |
| ***Roseburia*** | Correlation Coefficient | -0.064 | ,267^*^ | 0.181 | 0.130 | -0.080 | ,298^*^ | 0.199 | -0.216 | -0.197 | 0.236 |
|  | p-value | 0.600 | 0.024 | 0.132 | 0.320 | 0.538 | 0.019 | 0.121 | 0.094 | 0.129 | 0.064 |
| ***Ruminococcaceae_uc*** | Correlation Coefficient | -,246^*^ | ,526^**^ | ,438^**^ | 0.170 | 0.112 | ,263^*^ | 0.164 | 0.066 | -0.046 | 0.136 |
|  | p-value | 0.042 | 0.000 | 0.000 | 0.190 | 0.385 | 0.039 | 0.202 | 0.612 | 0.726 | 0.291 |
| ***Ruminococcus_g2*** | Correlation Coefficient | -,287^*^ | ,296^*^ | 0.228 | ,341^**^ | -0.124 | ,311^*^ | 0.032 | -0.092 | -0.093 | 0.032 |
|  | p-value | 0.017 | 0.012 | 0.056 | 0.007 | 0.336 | 0.014 | 0.803 | 0.480 | 0.478 | 0.807 |
| ***Ruminococcus_g4*** | Correlation Coefficient | -0.126 | ,406^**^ | ,389^**^ | ,294^*^ | 0.037 | 0.249 | 0.128 | 0.046 | -0.029 | 0.086 |
|  | p-value | 0.301 | 0.000 | 0.001 | 0.021 | 0.774 | 0.051 | 0.322 | 0.725 | 0.827 | 0.506 |
| ***Ruminococcus_g5*** | Correlation Coefficient | -0.122 | ,354^**^ | ,310^**^ | 0.218 | -0.027 | 0.188 | 0.066 | 0.204 | 0.236 | 0.135 |
|  | p-value | 0.319 | 0.002 | 0.009 | 0.092 | 0.835 | 0.143 | 0.612 | 0.114 | 0.067 | 0.295 |
| ***Serratia*** | Correlation Coefficient | -0.152 | 0.100 | 0.137 | ,293^*^ | 0.027 | ,251^*^ | 0.021 | 0.076 | 0.057 | -0.030 |
|  | p-value | 0.214 | 0.409 | 0.254 | 0.022 | 0.836 | 0.049 | 0.869 | 0.559 | 0.662 | 0.820 |
| ***Sporobacter*** | Correlation Coefficient | -,279^*^ | 0.192 | 0.121 | 0.108 | 0.008 | ,362^**^ | 0.116 | -0.082 | -0.090 | 0.114 |
|  | p-value | 0.020 | 0.109 | 0.316 | 0.407 | 0.953 | 0.004 | 0.369 | 0.531 | 0.489 | 0.379 |
| ***Streptococcaceae_uc*** | Correlation Coefficient | -0.167 | ,384^**^ | ,294^*^ | 0.116 | 0.021 | -0.024 | 0.029 | 0.095 | -0.022 | 0.012 |
|  | p-value | 0.171 | 0.001 | 0.013 | 0.371 | 0.869 | 0.853 | 0.824 | 0.468 | 0.869 | 0.923 |
| ***Streptococcus*** | Correlation Coefficient | -0.081 | ,247^*^ | 0.164 | -0.056 | -0.127 | -0.042 | -0.087 | -0.059 | -0.067 | -0.009 |
|  | p-value | 0.508 | 0.038 | 0.172 | 0.670 | 0.326 | 0.743 | 0.503 | 0.649 | 0.606 | 0.942 |
| ***Subdoligranulum*** | Correlation Coefficient | -0.090 | ,234^*^ | 0.159 | 0.023 | 0.019 | ,254^*^ | 0.225 | -0.128 | -0.203 | 0.192 |
|  | p-value | 0.460 | 0.049 | 0.185 | 0.862 | 0.886 | 0.046 | 0.079 | 0.327 | 0.116 | 0.135 |
| ***Veillonella*** | Correlation Coefficient | -0.140 | ,281^*^ | ,303^*^ | 0.072 | 0.208 | 0.193 | -0.059 | -0.158 | 0.097 | -0.109 |
|  | p-value | 0.253 | 0.018 | 0.010 | 0.580 | 0.106 | 0.132 | 0.649 | 0.224 | 0.458 | 0.397 |
